# Georeferencing sepsis in São Paulo city

**DOI:** 10.1186/cc10161

**Published:** 2011-06-22

**Authors:** E Silva, AS Cypriano, LF Lisboa, M Cendoroglo, F Colombari, BFC Santos

**Affiliations:** 1 Hospital Israelita Albert Einstein, São Paulo - SP, Brazil

## Introduction

Sepsis is a worldwide disease with heterogeneous outcome. The main factors related to prognosis are age, associated comorbidities, invader virulence, and time to therapeutic initiation. Data related to social-economical attributes have been scarcely investigated.

## Objective

To evaluate the distribution of sepsis-associated deaths in São Paulo city using a geographic information system (GIS); to verify whether there is any correlation between socioeconomic status and number of deaths.

## Methods

GIS is a system for input, storage, manipulation, and output of geographic information. GIS allows one to know the socioeconomic conditions of the region studied, including provision of health services, spatial data (rivers, parks, and so on), population data (age and sex), and estimated demand for health services. Thus, GIS could support health managers for planning, monitoring, priority setting and decision-making. Sepsis was identified through death certificates using several International Disease Codes including, but not restricted to, sepsis, septicemia, pneumonia, urinary tract infection, wound surgical infection, bloodstream infection, meningitis, and multiple organ failure among others.

## Results

**Figure 1:**
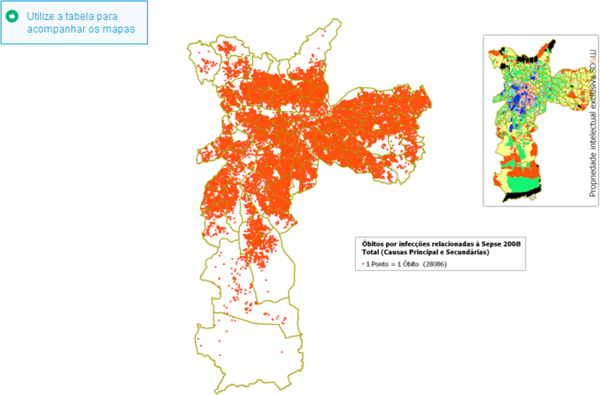


Figure 1 (overleaf) depicts every death according to the location of residence.

## Conclusion

Death secondary to sepsis is widely distributed throughout the regions of São Paulo, and further analysis needs to be done in different subgroups for better characterization and contrast of this syndrome in distinct regions and socioeconomic strata of the city.

